# Rheumatic Heart Disease in Europe: Insights from a Pilot Screening Study and a Scoping Review

**DOI:** 10.5334/gh.1573

**Published:** 2026-07-16

**Authors:** Martina Steinmaurer, Lea Baertz, Taariq Salie, Shekhar Saha, Dominik Joskowiak, Ulrich Seybold, Eimo Martens, Joselyn Rwebembera, Christian Hagl, Mark Engel

**Affiliations:** 1 Department of Internal Medicine I, Department of Clinical Medicine, TUM School of Medicine and Health, Technical University of Munich, Munich, Germany; 2 Department of Cardiac Surgery, LMU University Hospital, LMU Munich, Germany; 3 Cape Heart Institute, Department of Medicine, Faculty of Health Sciences, University of Cape Town, Cape Town, South Africa; 4 Center for Clinical Infectious Diseases and Department of Medicine IV, LMU University Hospital, LMU Munich, Germany; 5 Department of Adult Cardiology, Uganda Heart Institute, Kampala, Uganda; 6 Cochrane South Africa, South African Medical Research Council, Cape Town, South Africa

**Keywords:** Rheumatic Heart Disease, echocardiographic screening, World Heart Federation criteria, migrants, Europe, endemic regions

## Abstract

**Background:**

Rheumatic heart disease (RHD) remains a preventable cause of cardiovascular morbidity/mortality, disproportionately affecting socioeconomically disadvantaged populations. While largely controlled in high-income countries, RHD persists in low- and middle-income countries and may remain underrecognized among migrants from endemic regions in Europe, where systematic surveillance data are lacking. This study aimed to explore the implementation and diagnostic yield of echocardiographic screening in a migrant population and to contextualize these findings through a scoping review of European RHD research.

**Methods:**

We conducted a single-center pilot study in Munich, Germany, screening 150 recently-arrived migrants (aged 5–26 years) from RHD-endemic regions using 2023 World Heart Federation (WHF) echocardiographic criteria. Medical history was documented. Furthermore, we conducted a scoping literature review of European studies reporting RHD published since 2000, in accordance with Cochrane and PRISMA guidelines (PROSPERO ID: CRD42024538000).

**Results:**

Among screened participants (mean age 20.0 ± 5.9 years), ten (6.8%) showed abnormalities warranting confirmatory echocardiography according to the WHF protocol for RHD diagnosis, with mitral regurgitation being the most frequent finding. No participant had a prior diagnosis of RHD. Screening was integrated into routine health examinations without any obvious disruption of routine workflows. The scoping review identified 86 publications, predominantly from Turkey and Italy, highlighting fragmented research, methodological heterogeneity, limited prevalence data, and a focus on surgical management rather than early detection. No multicenter screening studies in asymptomatic at-risk populations were identified, limiting a more accurate assessment of disease burden.

**Conclusions:**

This study suggests that systematic echocardiographic screening for RHD in high-risk migrant populations in Europe may facilitate the identification of individuals with echocardiographic abnormalities requiring confirmatory evaluation. Combined with the fragmented European literature, these findings underscore the need for harmonized surveillance, larger multicenter studies, and careful evaluation of targeted screening approaches into migrant health programs to inform future epidemiologic and implementation research.

**Lay Summary:**

**What question did this study address, and why does it matter?**

This study asked whether echocardiographic screening for rheumatic heart disease (RHD) can be implemented in migrant health assessments in Europe, and whether such screening could contribute to a better understanding of RHD in a potentially high-risk population. In addition, we sought to explore whether existing research suggests that RHD may be under-recognized in selected high-risk populations in Europe, particularly in the context of migration from endemic regions. To address this, we reviewed the current European literature to assess the extent of research activity and available evidence on RHD.

**Where was the study done, and what were the main results?**

The study was conducted in Munich, Germany, where 150 recently-arrived migrants aged 5–26 years underwent echocardiographic screening as part of a pilot program. Approximately 7% of participants showed cardiac abnormalities that may require further evaluation, although these findings do not represent confirmed diagnoses. In addition, a scoping review of 86 publications from across Europe was performed to contextualize the pilot findings within the broader European RHD literature. The literature was highly fragmented, with most studies originating from a small number of countries. Many focused on hospital-based management rather than early detection, and only a few addressed screening in at-risk populations such as migrants.

**What do these findings mean in practice?**

Given that early identification of RHD could help prevent long-term cardiac complications, echocardiographic screening may be a relevant component of routine migrant health assessments to identify individuals requiring further diagnostic evaluation. RHD may still be present in select high-risk groups in Europe, but appears to be insufficiently systematically monitored. Since current European evidence remains limited and fragmented, we recommend that larger studies be conducted to determine whether targeted screening should be implemented more widely.

## Graphical Abstract

**Figure:**
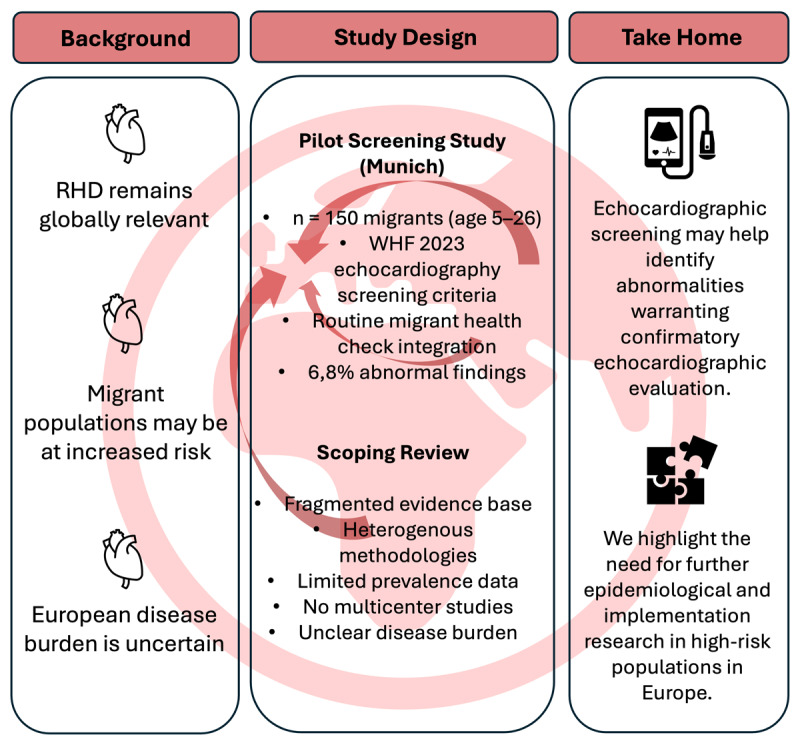


## Introduction

Rheumatic heart disease (RHD) remains a major cause of preventable cardiovascular morbidity and mortality worldwide, particularly affecting children, adolescents, and young adults from socioeconomically disadvantaged populations (1). RHD results from immune-mediated valvular damage following acute rheumatic fever (ARF), itself a complication of Group A Streptococcus (GAS) infection, including pharyngitis and, in certain settings, skin infections (2,3,4). Without adequate prevention, up to 60–65% of individuals with ARF develop chronic valvular heart disease (5,6,7). Timely diagnosis and administration of penicillin G, through secondary antibiotic prophylaxis (SAP), can effectively prevent disease progression (8).

RHD has become rare in most high-income countries (HICs) following major public health and healthcare improvements, but continues to affect indigenous populations in these settings (9,10) and remains highly prevalent in low- and middle-income countries (LMICs) (11,12,13,14,15,16). Global advocacy efforts by the World Heart Federation (WHF), the World Health Assembly, and the World Health Organization (WHO) have recently refocused attention on RHD as a neglected disease, emphasizing echocardiographic screening and early secondary prevention as key strategies to reduce disease burden (17,18,19,20,21).

In Europe, increasing migration from RHD-endemic regions has renewed concerns about the potential under-recognized burden of RHD. Countries with high RHD prevalence account for a substantial proportion of the European Union’s foreign-born population (22,23). Despite this demographic shift, RHD remains largely absent from European cardiovascular screening strategies, and awareness among healthcare providers is limited (24). The 2024 Global Burden of Disease analysis estimates that 54.8 million individuals worldwide are affected by RHD, with the greatest burden in LMICs with low Socio-demographic Index, suggesting that migrant populations in Europe may carry a disproportionately high, yet poorly documented, risk of subclinical disease (25).

To date, available literature on RHD in Europe appears to be derived from highly selected populations and is geographically concentrated in Turkey, while data from other European countries remain scarce. Against this background, isolated screening data are difficult to interpret and insufficient to estimate disease burden in at-risk populations. This study therefore combines two complementary components: a pilot echocardiographic screening study among recently arrived migrants in Germany to identify echocardiographic findings that warrant further evaluation, and a scoping review of European RHD research to contextualize these findings within existing epidemiologic and methodological evidence. Taken together, this paper reports the pilot cohort (n = 150), previously presented in part at the ESC Congress 2025 (26), provides an overview of current RHD research in Europe, highlights methodological heterogeneity and knowledge gaps, and discusses implications for future epidemiologic studies and targeted screening approaches.

## Methods

This study employed a dual-component design comprising a pilot echocardiographic screening study and a scoping review. The two components were designed to be complementary; the pilot study generated empirical screening data, while the scoping review provided contextualization within existing European RHD research.

### Pilot Screening Study

We conducted a single-center pilot screening study to explore the diagnostic yield of targeted echocardiographic screening for RHD in European migrant populations and its implementation within routine health examinations. Participants were recently arrived migrants from RHD-endemic countries who had resided in endemic regions for at least five years. Recruitment occurred during the mandatory health check-up following registration with the local Health Department in the metropolitan city of Munich, Germany.

Medical history was collected for all participants, including self-reported prior episodes of acute rheumatic fever (ARF), family history of ARF, and cardiovascular symptoms. Socioeconomic and educational indicators were collected descriptively using a questionnaire capturing housing conditions, household size, and educational level. While self-reported data are subject to recall bias and potential misclassification, they, provide preliminary insight into the participants’ prior health experiences. Transthoracic echocardiography was performed according to the 2023 WHF criteria for RHD. Exclusion criteria included known congenital heart disease, prior cardiac surgery, or inability to provide informed consent due to language barriers. Screening was performed by a medical doctoral student who had previously received structured training in focused echocardiography. Training included supervised acquisition of standard echocardiographic views and review of representative cases with an experienced physician. In cases of uncertainty, findings were discussed with the supervising physician during the screening phase. Echocardiography was performed in a focused screening setting rather than a full diagnostic echocardiographic laboratory environment.

The primary outcome was the proportion of participants with echocardiographic findings meeting predefined WHF screening criteria warranting confirmatory evaluation for possible RHD. In addition, the integration of echocardiographic screening into routine public health examinations was described. Ethical approval was obtained from the institutional ethics committee (Ethics Approval Number 24-0592), and the study was conducted in accordance with the Declaration of Helsinki. A detailed study protocol is available from the authors upon request.

### Scoping Review

A scoping literature review was conducted to inform interpretation of pilot screening findings in the absence of standardized prevalence benchmarks and to characterize the availability and structure of European RHD evidence. The review aimed to identify existing evidence, methodological gaps, and priorities for future European RHD initiatives. The review followed methodological guidance from the Cochrane Collaboration and PRISMA recommendations for scoping reviews (27,28) and was prospectively registered in PROSPERO (ID: CRD42024538000).

A comprehensive search strategy was developed to maximize sensitivity across electronic databases PubMed and Cochrane. Searches incorporated both free-text terms and database-specific controlled vocabulary (including Medical Subject Headings [MeSH]), adapted for each database individually. Search terms combined concepts relating to ‘rheumatic heart disease,’ ‘prevalence,’ and European populations/countries. The complete search strategy is provided in the Supplementary Material (Table S2).

Eligible studies included all English-language publications since 2000 reporting on RHD in European populations. The year 2000 was selected to reflect the beginning of a modern global health era characterized by intensified international public health collaboration and surveillance initiatives. Studies focusing exclusively on Group A streptococcal infection without RHD-related outcomes were excluded, as were narrative reviews, opinion articles, and case reports. Where duplicate publications of the same dataset were identified, only the most recent or comprehensive report was included.

**Figure 1:**
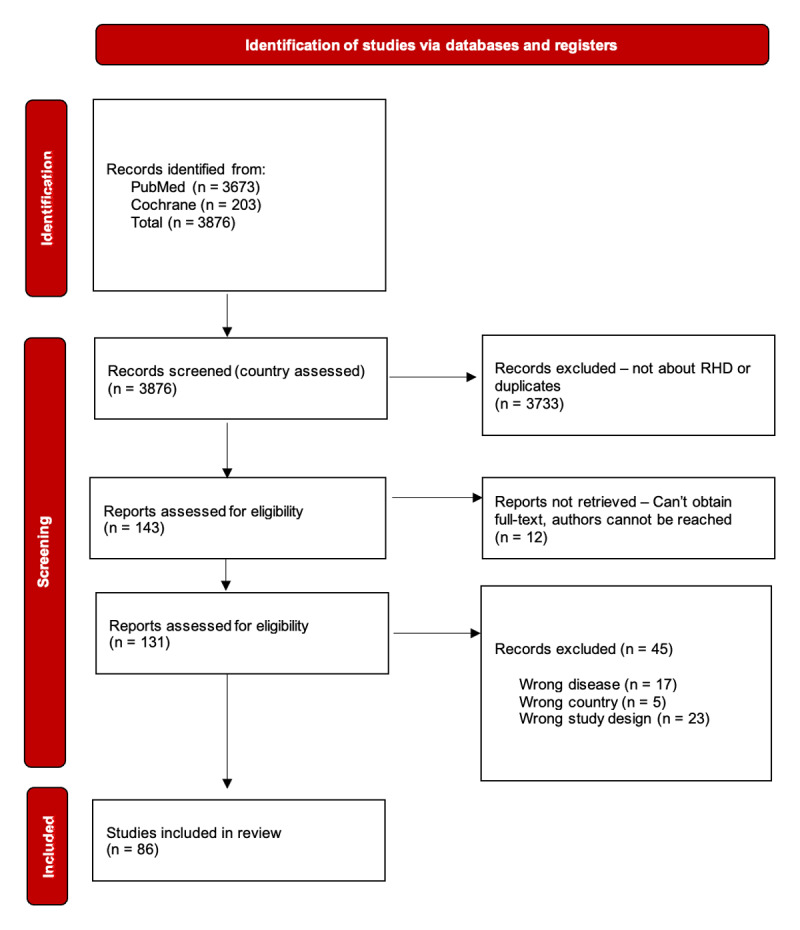
Flow diagram of the scoping review process. The diagram illustrates the identification, screening, eligibility assessment, inclusion, and exclusion of studies on rheumatic heart disease in Europe, following the PRISMA framework. Numbers of records at each stage are indicated, highlighting reasons for exclusion and the final set of included studies.

Titles, abstracts, and full texts were independently screened by two reviewers according to the PICO framework, with discrepancies resolved by consensus (Figure 1). Summary tables of included and excluded studies are provided in the Supplementary Material (Tables S3–S4), and key studies are summarized in Table 2. As this review was conducted as a scoping review intended to map the breadth and characteristics of the available literature rather than quantitatively synthesize outcomes, a formal risk-of-bias assessment was not performed. The review protocol additionally informed a subsequent meta-analysis of European RHD prevalence data (Steinmaurer et al., manuscript in preparation).

## Results

### Pilot Screening Study

A total of 150 participants aged 5-26 years (mean 20.0 ± 5.9 years) were screened. Of these, 40.7% were female and 59.3% male. Participants originated from 11 countries, predominantly Yemen (n = 31, 20.7%), Uganda (n = 25, 16.7%), and Sierra Leone (n = 24, 16%). Socioeconomic and educational indicators suggested generally adequate access to basic utilities and a wide range of educational levels.

Overall, ten participants (6.8%, 95% CI, 3.2–11.9%) met WHF criteria for findings warranting confirmatory evaluation, with mitral regurgitation being the most common abnormality. None of the participants with echocardiographic abnormalities had a prior diagnosis of RHD, and general awareness of ARF was low. Among all participants, 3.3% reported a personal history of rheumatic fever, and a similar proportion reported a family history. Other self-reported histories included high fever (25.3%), streptococcal infection (2%), carditis (14%), chorea (5.3%), non-itchy, stem-emphasized skin rash (6%), and severe joint pain (30.7%).

Participants with echocardiographic abnormalities were older than the overall cohort (median age 24 vs. 20 years) and predominantly male (80% vs 59% in the overall cohort). Most originated from African countries, notably Uganda (n = 4) and Tanzania (n = 2), consistent with the overall cohort composition (81/150 participants from African countries). However, no statistical comparisons were performed due to the small number of participants. Acceptability of the screening procedure was subjectively reported as good; however, no formal acceptability measures were collected. Application of the 2023 WHF criteria was practicable, though strict interpretation of indeterminate findings, especially mitral valve restriction, was necessary to avoid overdiagnosis. Further demographic and clinical details are provided in Table 1 and the Supplementary Material (Table S1).

**Table 1:** Demographic and clinical characteristics of the screened population and participants from our pilot study with echocardiographic abnormalities warranting confirmatory echocardiography.


CHARACTERISTIC	TOTAL POPULATION (n = 150)	ECHOCARDIOGRAPHIC ABNORMALITIES (n = 10)

**Sex**	Male: 59.3% (89) Female: 40.7% (61)	Male: 80% (8) Female: 20% (2)

**Age (years)**	Median: 20 (Range 5–26) Median Asia: 20.2 Median Africa: 20.6	Median: 24 (Range 19–26)

**Origin**	Africa: 54% (81) Asia: 46% (69)	Africa: 60% (6) Asia: 40% (4)

**Highest Education**	No schooling: 6 (Median age 9.5) Primary: 34 (Median age 13.5) Secondary: 50 (Median age 23) High school: 43 (Median age 23) University: 16 (Median age 25)	No schooling: 0 Primary: 1 (22) Secondary: 2 (19, 26) High school: 4 (23, 24, 25, 25) University: 3 (22, 25, 26)

**Housing Conditions**	Multiple rooms + water + electricity: 83 1 room + water + electricity: 24 Electricity only: 12 Water only: 17 None: 13	Multiple rooms + water + electricity: 6 1 room + water + electricity: 1 Electricity only: 2 Water only: 1 None: 0

**Previous RHD-Associated Conditions**	Rheumatic fever: 3.3% Cardiac issues: 14% Chorea: 5.3% Skin rash: 6% Joint problems: 30.7%	Rheumatic fever: 0% Cardiac issues: 10% (1/10) Chorea: 0% Skin rash: 30% (3/10) Joint problems: 50% (5/10)

**Echocardiographic Findings**	Not applicable	Mitral Regurgitation > 2 cm: 9/10 Aortic Regurgitation: 1/10


Screening examinations were estimated to require approximately 3–10 minutes per participant, and informal feedback from the participating health authority suggested no major perceived disruption of routine examinations. Approximately 12 individuals declined participation, most commonly because they perceived themselves as healthy or were unfamiliar with RHD. Language barriers were an infrequent reason for non-participation, as participants occasionally used ad hoc translation support via personal mobile devices on their own initiative. Screening was conducted by a medical doctoral student under the supervision of a physician, reflecting a minimal staffing setup typical for pilot implementation in routine clinical environments. These observations suggest that echocardiographic screening may be implementable within routine health check-ups and could potentially be integrated into similar clinical settings.

### Scoping Review

Our review identified 86 publications addressing RHD research in Europe since 2000. Research activity was highly fragmented, with most studies originating from Turkey (n = 43), followed by Italy (n = 10), and fewer studies from France, Greece, Germany, and other countries. Turkey represented a distinct context, with both high research output and a persistently elevated RHD burden. Studies spanned epidemiology, maternal health, and molecular/immunogenetic research. Most other European studies focused on registries, surgical and interventional management, and clinical outcomes, with RHD rarely being the primary focus.

Notably, studies from Italy report measurable rates of clinical and subclinical RHD, suggesting a potential burden of disease in selected high-risk populations. For example, Grimaldi et al. reported that RHD accounted for 41% of cardiac surgical indications among migrants referred for surgery in an Italian center between 2003 and 2011, highlighting the continued clinical relevance of RHD in migrant populations (29). Similarly, Condemi et al. identified a prevalence of borderline and definite RHD of 18.7% and 2.6%, respectively, in a screened healthy migrant pediatric and young adult population in Italy, underscoring the need for further epidemiological assessment in these groups (30).

Additional European data further support the heterogeneity of RHD epidemiology. Registry and survey data, including the Euro Heart Survey and national valve registries, indicate that while degenerative valve disease predominates overall, rheumatic etiology remains relevant in specific valve lesions. In the Turkish registry, RHD accounted for 46% of valvular heart disease cases, whereas in the Euro Heart Survey, rheumatic involvement remained substantial in mitral stenosis (85.4%) and present across other valve lesions (31,32).

Mortality analyses from high-income countries have demonstrated overall declining trends in RHD-related mortality between 2000 and 2020, although with persistent or atypical patterns in certain settings, including Germany, highlighting ongoing uncertainty in disease dynamics (33).

Thematic categorization revealed publications addressing surgical/interventional research (29,34,35,36,37,38,39,40,41,42,43,44,45,46,47,48), studies of other cardiovascular conditions, including RHD populations (49,50,51,52,53,54,55,56,57,58,59,60,61,62,63,64,65,66,67,68,69,70), epidemiology (30,32,33,71,72,73,74,75,76,77,78,79,80), and fundamental research (81,82,83,84,85,86,87). Additional studies addressed diagnostic questions (88,89,90,91,92,93,94,95,96,97,98,99,100,101,102,103), pregnancy outcomes (104,105,106,107), and modelling or registry-based epidemiology, including Global Burden of Disease analyses (22,25,108,109,110,111) and the Valvular Heart Disease Registry (112). A selection of representative publications is summarized in Table 2.

**Table 2:** Selected key publications from the scoping review on rheumatic heart disease in Europe. These studies were chosen based on their relevance to the RHED-MIG study objectives, including epidemiology, clinical characteristics, diagnostics, and insights into high-risk populations


REFERENCE ID	TITLE	AUTHOR/YEAR	STUDY TYPE	POPULATION/COUNTRY	METHOD	CONCLUSION

https://doi.org/10.2459/JCM.0000000000000228	Heart surgery for immigrants in Italy: burden of cardiovascular disease, adherence to treatment and outcomes	Grimaldi et al. 2016	prospective/observational	Migrants needing heart surgery in Italy	A clinical and echocardiographic survey was conducted on 154 consecutive immigrants referred for heart surgery to San Raffaele Hospital in Milan between 2003 and 2011.	The major cause of heart disease was RHD (41%). Cardiovascular diseases represent a major health topic among immigrants in developed countries. RHD still is the predominant cause of hospitalization for heart surgery, with nonrheumatic valvulopathies and IHD being the second and third causes, respectively. The data underlines the need to reinforce prevention and care strategies in the matter of immigrant health and warrants the urgent attention of the international public health and research communities.

https://doi.org/10.5543/tkda.2013.71430	The Turkish registry of heart valve disease	Demirbag et al. 2013	prospective	Patients with VHD admitted to the cardiology clinics in 33 cities from seven geographical regions in Turkey were included in the study	Informed Data was collected via the internet from each hospital. The dataset comprised around 200 different parameters, such as demographical variables like age, gender, education, and the number of children, background, symptoms, co-morbidity risk factors, affected valves, etiologies, electrocardiographic and echocardiographic findings, and suggested treatments. The etiologies of VHD were classified according to history, as well as clinical and echocardiographic findings.	This study showed that the main cause of VHD is rheumatic fever. Mitral regurgitation and multiple valvular lesions are the most frequent VHDs in Turkey. Percutaneous mitral balloon valvuloplasty and mechanical prosthetic valve replacement are the preferred treatment methods for VHD.

https://doi.org/10.1007/s00392-023-02168-6	Age period cohort analysis of rheumatic heart disease in high-income countries	Hibino et al. 2023	secondary data analysis (Age–Period–Cohort modelling)	General population in selected high-income countries, based on national health, mortality, or disease registries and global epidemiological datasets	The WHO mortality database was analyzed to determine trends in mortality from rheumatic heart disease in the UK, Germany, France, Italy, Japan, Australia, the USA, and Canada from 2000 to 2020. Age-cohort-period modeling was used to estimate cohort and period effects. Net drift (overall annual percentage change), local drift (annual percentage change in each age group), and heterogeneity were calculated.	Mortality trends from rheumatic heart disease were decreasing in the study high-income countries, except for Germany where higher mortality and two peaks in annual percentage change in younger and older age groups warrant further investigation.

https://doi.org/10.1186/s12969-019-0314-9	Screening of asymptomatic rheumatic heart disease among refugee/migrant children and youths in Italy	Condemi et al. 2019	prospective/observational	Migrant populations in Italy	From February 2016 to January 2018, Médecins Sans Frontières conducted a weekly mobile screening by echocardiography in reception centers and family houses for unaccompanied foreign minors in Rome, followed by fix echocardiographic retesting for those resulting positive at screening. ‘Definite’ and ‘borderline’ cases were defined according to the World Heart Federation criteria.	Screening for RHD among the unaccompanied migrant minors in Italy proved to be feasible. The burden of ‘definite RHD’ was similar to that identified in resource-poor settings, while the prevalence of ‘borderline’ cases was higher than reported in other studies. In view of these findings, the health system of high-income countries, hosting migrants and asylum seekers, are urged to adopt screening for RHD particular among the silent and marginalized population of refugee and migrant children.

https://doi.org/10.1017/S1047951119002075	Echocardiographic screening for rheumaticheart disease in Turkish schoolchildren	Atalay et al. 2019	prospective/multicenter	Schoolchildren in Turkey	This study aimed to investigate the prevalence of subclinical rheumatic heart disease in schoolchildren aged 5–18 by using portable echocardiography in Ankara, Turkey. The echocardiography screening was performed by a pediatric cardiologist for all the cases. Echocardiographic studies were assessed according to 2012 World Heart Federation criteria for RHD.	The frequency of rheumatic heart disease was found to be 15/1000, a finding similar to those of recent echocardiographic screening studies performed in middle and high-risk populations. It was concluded that to decrease the burden of RHD, echocardiographic screening studies are necessary, and long-term follow-up of children with echocardiographically diagnosed subclinical rheumatic heart disease is needed.

https://pubmed.ncbi.nlm.nih.gov/19301555	The spectrum of rheumatic heart disease in the southeastern Anatolia endemic region: results from 1900 patients	Ozer at al. 2009	retrospective	People presenting for echocardiography examination at a cardiology laboratory in the Southeastern Anatolia region in Tukey	In this retrospective study, transthoracic echocardiographic data acquired between June 2003 and January 2008 were reviewed. Information was gathered from the database of the authors’ echocardiography laboratory, which included age, gender, clinical diagnosis, and echocardiographic findings. In patients with more than one echocardiographic record, only the first echocardiographic data were included in the study.	A first admission with full echocardiographic data of RHD was found in 1,900 cases among 43,900 subjects screened (4.3%). Currently, RHD remains an alarming and unresolved health problem in the Southeastern Anatolia region. While almost 75% of affected subjects were female, males were more severely affected. In addition, subjects were relatively old, and most were affected by mixed valvular disease of an advanced stage.

https://doi.org/10.1007/s00404-009-1050-z	Pregnancy outcomes in women with heart disease	Madazli et al. 2010	retrospective	Pregnant women with cardiac disease in Turkey	A retrospective analysis was carried out of 144 pregnancies in women with cardiac disease who delivered at Cerrahpaşa Medical Faculty in Istanbul between 1997 and 2006. Perinatal and maternal outcomes were interpreted according to the type of heart disease and status of the patient according to the New York Heart Association (NYHA) classification.	The rate of RHD was 87.5%. Rheumatic heart disease with pregnancy is still predominant in Turkey. Most of the patients were in a good functional group. Maternal morbidity strongly correlates with maternal cardiac classification.

https://doi.org/10.14744/AnatolJCardiol.2019.55267	Screening and evaluation of newly diagnosed cardiovascular diseases in first-trimester asymptomatic pregnant women in a tertiary antenatal care center in Turkey	Bozkaya et al. 2020	prospective	Pregnant women without cardiac disease in Turkey	A total of 900 women in the first trimester of pregnancy, who attended the antenatal outpatient clinic of a tertiary care center in Ankara, Turkey, for a routine pregnancy examination, were recruited into this prospective study. Patients with a history of chronic systemic diseases, cardiovascular disease, and/or a family history of an early onset cardiovascular disease, and multiple pregnancies were excluded. Patients who were included in the study underwent electrocardiography and transthoracic echocardiography by the same cardiologist.	A cardiovascular disease rate of 5.2% was observed among healthy women in the first trimester of pregnancy, of whom 55.3% had RHD. These results show that clinicians must keep in mind that during pregnancy, physiological changes in the cardiovascular system may aggravate an undiagnosed disease, and they should be alert even in case of mild cardiac symptoms that may interfere with pregnancy complaints.

https://doi.org/10.1161/CIRCULATIONAHA.117.032561	Pregnancy outcomes in women with rheumatic mitral valve disease results from the registry of pregnancy and cardiac disease	van Hagen et al. 2018	observational registry study	Pregnant women with cardiac disease from emerging countries	The Registry of Pregnancy and Cardiac Disease is an international prospective registry, and consecutive pregnant women with cardiac disease were included. Pregnancy outcomes in all women with rheumatic mitral valve disease and no pre-pregnancy valve replacement were described in the study (n = 390). A maternal cardiac event was defined as cardiac death, arrhythmia requiring treatment, heart failure, thromboembolic event, aortic dissection, endocarditis, acute coronary syndrome, and hospitalization for other cardiac reasons or cardiac intervention. Associations between patient characteristics and cardiac outcomes were checked in a 3-level model (patient–center–country).	RHD was diagnosed in 380 patients. Mortality was 1.9% during pregnancy. Around 50% of the patients with severe rheumatic MS and 23% of those with significant MR developed heart failure during pregnancy. Pre-pregnancy counseling and considering mitral valve interventions in selected patients are important to prevent these complications.


Taken together, these findings suggest that while RHD has substantially declined in Europe, it may remain under-recognized in selected high-risk populations among migrants from endemic regions. However, available evidence is largely derived from hospital-based, registry-based, or highly selected cohorts, limiting inference on population-level trends.

## Discussion

This study comprises two distinct components: a pilot echocardiographic screening component and a scoping review. While conducted in parallel, both components were designed to address complementary aspects of RHD research in Europe: to assess the yield of WHF-based echocardiographic screening in a European migrant health setting, and to contextualize these findings within the broader and highly heterogeneous European RHD literature. This pilot study identified echocardiographic findings warranting confirmatory evaluation for possible RHD in 6.8% of screened participants amongst migrants in a European metropolitan setting, while demonstrating that WHF-based echocardiographic screening could be implemented within a routine migrant health setting under pilot conditions. Importantly, the scoping review was not intended to estimate prevalence but to characterize methodological gaps and the absence of standardized epidemiological data, which limits the interpretation of these screening-based findings. Together, both components highlight the current lack of structured surveillance frameworks for RHD in Europe and support the rationale for targeted screening studies in defined high-risk populations.

Migrant populations from RHD-endemic regions constitute a particularly vulnerable group, possibly harboring subclinical disease that remains undetected without targeted echocardiographic screening. Population-based echocardiographic screening in endemic regions has historically revealed a considerable burden of previously unrecognized RHD. In Uganda, echocardiographic screening of school-aged children detected definitive RHD at a rate of 1.48% (113), identifying three times as many cases as auscultation alone.

European data from Condemi et al. (30) among unaccompanied migrant minors in Italy already reported a high burden of echocardiographic abnormalities, with borderline RHD identified in up to 18% of participants. However, the vast majority of borderline RHD (93.4%) were classified based on isolated mitral valve morphological features. Definite RHD was observed in approximately 2.6% of the cohort. Consequently, the overall proportion of screening-positive findings approached 50%, largely driven by the high frequency of borderline classifications attributable to mitral valve morphology rather than hemodynamically significant valvular disease. In contrast, no cases in our cohort were classified as abnormal on the basis of mitral valve morphology alone; all were associated with demonstrable valvular insufficiency. These findings highlight the inherent challenges of interpreting echocardiographic screening results, where morphological variants may substantially influence screening positivity rates. Importantly, the terminology of ‘borderline’ and ‘definite’ RHD refers to the 2012 WHF echocardiographic criteria, which have since been updated; the 2023 WHF criteria no longer use this classification framework. Differences between the 2012 and 2023 WHF echocardiographic criteria may limit comparability with earlier European screening studies, particularly those relying on morphology-based ‘borderline’ classifications. In addition, modifications in the 2023 framework may further influence screening positivity rates and should be considered when interpreting historical prevalence estimates. Nevertheless, both studies indicate that echocardiographic abnormalities meeting WHF criteria can be detected in migrant populations residing in Europe and highlight the need for cautious interpretation and further epidemiological work in high-risk groups.

Abnormal echocardiographic findings should not be equated with clinically significant RHD requiring long-term secondary antibiotic prophylaxis or specialized cardiology follow-up. WHF-based screening criteria are designed to maximize sensitivity for early detection and may therefore identify valvular abnormalities of uncertain clinical significance. Although our cohort consisted of individuals originating from RHD-endemic regions, interpretation of screening findings within a European healthcare context remains challenging due to the absence of standardized epidemiological reference data. Consequently, some detected abnormalities may not represent clinically meaningful disease, highlighting the need for cautious interpretation of screening-based findings in migrant populations.

The higher median age of participants with echocardiographic abnormalities in our cohort reflects the known latency between initial Group A Streptococcus exposure and clinically manifest valvular disease, a pattern also observed in other screening contexts. The scoping literature review demonstrated that no standardized epidemiological framework for RHD surveillance exists in Europe. Relatively large-scale and methodologically structured screening programs conducted in endemic countries consistently demonstrate a substantial burden of latent disease when WHF criteria are applied. Only in Turkey have multicenter initiatives systematically evaluated subclinical disease across representative high-risk populations. We show that European RHD research is highly fragmented, with limited prevalence data, scarce early detection studies, and a strong focus on late-stage disease or surgical outcomes. Still, our data do not allow conclusions regarding population-level incidence or temporal trends in Europe.

Beyond diagnostic considerations, echocardiographic screening in migrant populations raises important ethical and implementation challenges. In the present pilot study, participants with screening-positive findings were provided with written recommendations for cardiology follow-up, as confirmatory echocardiography was not available within the study framework. While recently arrived migrants in Germany generally have access to basic healthcare services, navigation of the healthcare system, language barriers, continuity of care, and uncertainty regarding coverage of specialist investigations may nevertheless affect access to follow-up evaluation.

Taken together, the pilot screening data and scoping review provide complementary insights: the screening component identified abnormal echocardiographic findings warranting confirmatory echocardiography in a subset of participants, while the review demonstrates that the absence of harmonized epidemiological data limits meaningful benchmarking of these findings against a European baseline. These findings suggest that harmonized, multicenter studies may be helpful to more accurately quantify RHD burden, identify at-risk populations, and inform evidence-based public health strategies tailored to European contexts. Such efforts should build upon established RHD detection frameworks and consider integration into migrant health programs, alongside strengthened awareness and education initiatives.

## Strengths and Limitations

The strengths of this combined analysis include the integration of primary pilot data with a review of the European literature, providing both empirical and contextual insights. Still, our findings should be interpreted with caution, given the exploratory nature of the study, the absence of confirmatory diagnostic testing, and the potential for misclassification. Confidence intervals should be considered when interpreting this estimate. Feasibility was not formally assessed using predefined implementation metrics; accordingly, implementation conclusions are based on descriptive and subjective observations from the study setting. Echocardiographic screening was performed by a single operator, and no independent blinded over-reading of all examinations was undertaken. Participants were recruited exclusively during mandatory health examinations in a single metropolitan migrant health center in Munich. Our results are therefore limited by the single-center design and modest sample size. The population may not be representative of broader migrant populations across Germany or Europe when considering country of origin, migration trajectories, socioeconomic conditions, healthcare access, or underlying RHD risk. The scoping review is similarly constrained by heterogeneity in study designs, populations, outcome reporting, and variable application of diagnostic criteria across studies.

## Additional File

The additional file for this article can be found as follows:

10.5334/gh.1573.s1 Supplementary Material. Tables S1 to S4.

## Data Availability

Sociodemographic, clinical, and echocardiographic data from the pilot phase are provided in the Supplementary Material.
