# Supplementary material for: Rheumatic Heart Disease in Europe: Insights from a Pilot Screening Study and a Scoping Review
Source: Glob Heart. 2026 Jul 16;21(1):56. doi: 10.5334/gh.1573 (PMC13378423; doi:10.5334/gh.1573)
Supplement: Supplementary Material. — Tables S1 to S4. [file gh-21-1-1573-s1.pdf]

Country	Total Population (n=150)	Screen-Positive (n=10)
Uganda	25	4
Nigeria	8	1
Sierra Leone	24	1
Jemena	31	2
Afghanistan	17	0
Kongo	14	1
Tanzania	5	2
Myanmar	5	0
Syria	3	0
Pakistan	1	0
Mali	1	0

Table S1 Country of origin of the screened population and participants with echocardiographic abnormalities of our pilot study.

PubMed	("Rheumatic Heart Disease"[Mesh] OR "rheumatic heart disease" OR "RHD")<br>AND<br>("Prevalence"[Mesh] OR prevalence OR epidemiology OR incidence OR diagnosis)<br>AND<br>("Europe"[Mesh] OR Europe OR European Union OR Albania OR Andorra OR Armenia OR Austria OR Azerbaijan OR Belarus OR Belgium OR Bosnia OR Herzegovina OR Bulgaria OR Croatia OR Cyprus OR Czech Republic OR Denmark OR Estonia OR Finland OR France OR Georgia OR Germany OR Greece OR Hungary OR Iceland OR Ireland OR Italy OR Kazakhstan OR Kosovo OR Latvia OR Liechtenstein OR Lithuania OR Luxembourg OR Malta OR Moldova OR Monaco OR Montenegro OR Netherlands OR "North Macedonia" OR Norway OR Poland OR Portugal OR Romania OR Russia OR Serbia OR Slovakia OR Slovenia OR Spain OR Sweden OR Switzerland OR Turkey OR Ukraine OR "United Kingdom")<br><br>Filters: English language, publication year ≥2000
Cochrane Library	A simplified version of the above strategy using key terms “rheumatic heart disease” AND “Europe” AND “prevalence” was applied.
Google Scholar	A supplementary citation search was performed using combinations of: “rheumatic heart disease Europe prevalence”, “echocardiographic screening RHD Europe”, and “migrants rheumatic heart disease Europe”.

Table S2 Search Strategy: A systematic literature search was conducted in PubMed and the Cochrane Library. In addition, Google Scholar was screened for supplementary citation tracking purposes (non-systematic). The last search was performed in [03/2026].
The search combined controlled vocabulary (Medical Subject Headings [MeSH]) and free-text terms related to rheumatic heart disease and epidemiology. To ensure comprehensive coverage of European studies, both a general European filter and country-specific terms were applied.

**Surgical and interventional studies**				
1	Aphram et al. 2018	Eur J Cardiothorac Surg	Journal article	retrospective
2	Desnos et al. 2019	J Am Heart Assoc	Journal article	retrospective
3	Verdonk et al. 2018	Arch Cardiovasc Dis	Journal article	retrospective
4	Fabi et al.2021	Int J Cardiol	Journal article	retrospective
5	Grimaldi et al. 2016	J Cardiovasc Med	Journal article	prospective/observational
6	Augestad et al. 2000	Scand Cardiovasc J	Journal article	retrospective
7	Fedakar et al. 2010	Heart Surg Forum	Journal article	retrospective/comparative
8	Mavioglu et al. 2001	J Heart Valve Dis	Journal article	Prospective/observational
9	Tekumit et al. 2010	J Card Surg	Journal article	prospective
10	Yilmaz et al. 2004	Anadolu Kardiyol Derg	Journal article	retroseptive
11	Ismeno et al. 2001	Acta Cardiol	Journal article	prospective/observational
12	Karakaya et al. 2006	J Am Soc Echocardiogr	Journal article	prospective
13	Martens et al. 2011	Eur J Cardiothorac Surg	Journal article	prospective/multicenter
14	Tokmakoglu et al 2001	J Heart Valve Dis	Journal article	prospective
15	Amador et al. 2025	Monaldi Archives for Chest Disease	Journal article	retrospective
16	Gencoglu et al. 2024	BMC Cardiovascular Disorders	Journal article	retrospective
**Studies including RHD Patients within other disease cohorts**				
17	Landgren et al. 2006	Blood	Journal article	case-control

18	Eriksson et al. 2013	Am J Hum Biol	Journal article	retrospective
19	Le Ven et al. 2016	PLoS One	Journal article	retrospective
20	Taddio et al. 2020	Clin Exp Rheumatol	Journal article	retrospective/multicenter
21	Leszek et al. 2010	Clin Physiol Funct Imaging	Journal article	prospective
22	Goodman et al. 2015	Eur J Prev Cardiol	Journal article	prospective, population-based birth cohort
23	Odemis et al. 2006	J Natl Med Assoc	Journal article	prospective
24	Tavli et al. 2008	Pediatr Int	Journal article	prospective
25	Atalar et al. 2012	Coron Artery Dis	Journal article	retrospective/comparative
26	Mataraci et al. 2010	Heart Lung Circ	Journal article	prospective
27	Sucu et al. 2010	Turk Kardiyol Dern Ars	Journal article	retrospective
28	Bozbas et al. 2004	Anadolu Kardiyol Derg	Journal article	retrospective
29	Tugcu et al 2009	Turk Kardiyol Dern Ars	Journal article	retrospective
30	Sahin et al. 2016	Int J Pediatr Otorhinolaryngol	Journal article	prospective
31	Akram et al. 2009	Eur J Echocardiogr	Journal article	retrospective/comparative
32	Naveen et al. 2014	Int J Med Microbiol	Journal article	prospective
33	Callegari et al. 2020	Circ Arrhythm Electrophysiol	Journal article	retrospective/observational
34	Kranidis et al. 2000	Pacing Clin Electrophysiol	Journal article	prospective
35	Bilge et al. 2000	Jpn Heart J	Journal article	prospective
36	Temur et al. 2024	Niger J Clin Pract	Journal article	comparative
37	Watt et al. 2015	Am J Trop Med Hyg	Journal article	comparative
38	Benz et al. 2019	CJC	Journal article	registry/prospective
**Studies with an epidemiological focus**				
39	Demirbag et al. 2013	Arch Turk Soc Cardiol	Journal article	prospective
40	Hibino et al. 2023	Clin Res Cardiol	Journal article	database
41	Condemi et al. 2019	Pediatr Rheumatol Online J	Journal article	prospective/observational
42	Ascione et al. 2004	Int J Cardiovasc Imaging	Journal article	retrospective/prospective
43	Toledano et al. 2012	J Heart Valve Dis	Journal article	prospective
44	Atalay et al. 2019	Cardiol Young	Journal article	prospective/multicenter
45	Uner et al. 2009	Anadolu Kardiyol Derg	Journal article	prospective
46	Ozer at al. 2009	Heart Valve Dis	Journal article	retrospective
47	Phillips et al. 2017	Int J Epidemiol	Journal article	comparative
48	Yildiz et al. 2016	EHJ	Journal article	observational
49	Licciardiet al. 2025	Pediatric Cardiology	Journal article	retrospective
50	İrdem et al. 2024	Cardiology in the Young	Journal article	prospective
51	Ulkersoy et al. 2025	Pediatric Cardiology	Journal article	retrospective
**Basic and Translational Research on RHD**				
52	Gumus et al. 2018	Rheumatol Int	Journal article	observational cross-sectional
53	Stanevicha et al. 2003	Arthritis Res Ther	Journal article	Observational cross-sectional genetic association study
54	Simsek et al. 2011	Clin Rheumatol	Journal article	Observational genetic association (case-control)
55	Düzgün et al. 2007	Clin Rheumatol	Journal article	Case-control genetic association study
56	Gündogdu et al. 2007	J Heart Valve Dis	Journal article	Observational genetic association study
57	Polat et al. 2015	Acta Cardiol	Journal article	observational / cross-sectional
58	Atalar et al. 2003	J Heart Valve Dis	Journal article	Observational genetic association (case–control)
**RHD Diagnostic Research**				
59	Zamorano et al. 2004	J Am Coll Cardiol	Journal article	prospective/multicenter
60	Karpuz et al. 2017	Cardiol Young	Journal article	prospective
61	Yavuz et al. 2008	Eur J Pediatr	Journal article	Observational cohort / longitudinal follow-up
62	Ozdemir et al. 2014	Pediatr Cardiol	Journal article	prospective
63	Ucar et al. 2011	J Heart Valve Dis	Journal article	prospective/comparative
64	Mutlu et al. 2003	Int J Cardiol	Journal article	prospective/comparative
65	Yıldırımtürk et al. 2013	Anadolu Kardiyol Derg	Journal article	observational
66	Dogan et al. 2006	J Am Soc Echocardiogr	Journal article	prospective
67	Tunaoğlu et al 2004	Anadolu Kardiyol Derg	Journal article	prospective
68	Alp et al. 2014	Pediatr Int	Journal article	prospective
69	Yavrum et al. 2023	North Clin Istanb	Journal article	retrospective
70	Kelmendi et al. 2009	Med Arh	Journal article	retrospective
71	Bal et al. 2024	Turk J Pediatr	Journal article	comparative
72	Poyraz et al. 2024	Int J Cardiovasc Imaging	Journal article	prospective
73	Sonsöz et al. 2025	Lipids	Journal article	comparative
74	Göbüt et al. 2025	Postgrad Med	Journal article	prospective/comparative
**RHD and pregnancy**				
75	Madazli et al. 2010	Arch Gynecol Obstet	Journal article	retrospective
76	Bozkaya et al. 2020	Anatol J Cardiol	Journal article	prospective
77	Iscan et al. 2006	J Heart Valve Dis	Journal article	retrospective

78	van Hagen et al. 2018	Circulation	Journal article	registry/prospective
**Global and Public Health Perspectives on RHD**				
79	White et al. 2018	Eur Heart J	Policy statement	guideline / consensus document
80	Watkins et al. 2017	N Engl J Med	Journal article	Epidemiological modelling / secondary data analysis
81	Scheel et al. 2021	Int J Cardiol	Journal article	observational
82	Oldgren et al. 2014	Circulation	Journal article	prospective
83	Chen et al. 2020	J Glob Health	Journal article	Observational, modelling study
84	Chen et al. 2024	J Am Heart Assoc	Journal article	retrospective, secondary, model-based data
85	Elmusharaf et al. 2025	BMJ Glob Health	Journal article	retrospective, secondary, model-based data
86	Abdelhamid et al. 2025	European Heart Journal	Journal article	prospective, register

Table S3 Included studies from the scoping review, organized by research focus.

**Studies not addressing RHD**				
1	Rimoin et al. 2005	Arch Dis Child	Journal article	prospective/comparative
2	Pazdernik et al. 2009	J Heart Valve Dis	Journal article	case series
3	Kafetzis et al. 2005	Eur J Clin Microbiol Infect Dis	Journal article	retrospective
4	Giannoulia-Karantana et al. 2001	Acta Paediatr	Journal article	retrospective
5	Breda et al. 2012	J Pediatr	Journal article	retrospective/comparative
6	Grassi et al. 2009	Clin Exp Rheumatol	Journal article	retrospective
7	Breda et al. 2013	Rheumatology (Oxford)	Journal article	comment
8	Minola et al. 2005	Infez Med	Journal article	retrospective/comparative
9	Gürses et al. 2021	J Paediatr Child Health	Journal article	prospective/multicenter
10	Erdem et al. 2020	Cardiol Young	Journal article	retrospective
11	Ozdemir et al. 2014	Tex Heart Inst J	Journal article	retrospective
12	Sahin et al. 2012	Turk J Pediatr	Journal article	prospective? Comparative
13	Güler at al. 2020	Cardiol Young	Journal article	retrospective
14	Olgunturk et al. 2006	Int J Cardiol	Journal article	retrospective/comparative
15	Polat et al. 2006	Cardiol Young	Journal article	prospective
16	Turhan et al. 2002	Am J Cardiol	Journal article	prospective
17	Papasavva et al. 2106	BMC Res Notes	Journal article	unclear
**Studies with a focus outside of Europe**				
18	Deepak et al. 2016	J Cardiothorac Vasc Anesth	statement	retrospective/comparative
19	Hunter et al. 2023	Heart	Journal article	retrospective/comparative
20	Hunter et al. 2021	Echocardiography	Journal article	unclear
21	Marijon et al. 2009	Circulation	Journal article	unclear
**Studies with an inappropriate study design**				
22	Poitras	Arch Environ Health	Journal article	editorial
23	Cheng et al 2002	Am J Cardiol	Journal article	comment
24	Vesslova et al. 2004	Cardiol Young	Journal article	letter to the editor
25	Sliwa et al. 2018	Eur Heart J	Journal article	comment
26	Zorkun et al. 2013	Anadolu Kardiyol Derg	Journal article	letter to the editor
27	Kelly et al. 2014	BMJ Case Rep	Journal article	case report
28	Lynskey et al. 2011	Curr Opin Infect Dis	Journal article	review
29	McGlacken-Byrne et al. 2015	BMJ Case Rep	Journal article	case report
30	Rosenthal et al. 2013	Am J Cardiol	Journal article	editorial
31	Silverman et al. 2000	Am J Cardiol	Journal article	obituary
32	Ormerod et al. 2006	J Med Biogr	Journal article	obituary
33	Kadir et al. 2004	Ann Thorac Surg	Journal article	case report
34	Wrong et al. 2003	J R Soc Med	Journal article	obituary
35	Young et al. 2016	Br J Hosp Med (Lond)	Journal article	editorial
36	Medina et al. 2002	Indian Heart J	Journal article	review
37	Deshpande et al. 2002	J Postgrad Med	Journal article	obituary
38	Berry et al. 2007	Circulation	Journal article	editorial
39	Gewitz et al. 2015	Circulation	Statement	Statement
40	Ferrieri et al. 2002	Circulation	Journal article	Guidline
41	Aouba et al. 2004	Presse Med	Journal article	case report
42	de Almeida et al. 2024	Rev Port Cardiol	Letter to the Editor	Unclear
43	Bray et al. 2024	Cochrane Database Syst Rev	Meta Analyses	Unclear
44	Hammer at al. 2024	Cardiovasc Drugs Ther	comment	unclear
45	Ferrieri et al. 2002	Circulation	Journal article	Guidline

Table S4 Excluded studies from the scoping review, categorized by reason for exclusion
